# Views on and experiences with medicinal cannabis among Canadian veterans who live with pain: A qualitative study

**DOI:** 10.1080/24740527.2023.2232838

**Published:** 2023-09-08

**Authors:** David P. Storey, Natalie R. Keeler-Villa, Nick Harris, Jennifer Anthonypillai, Gregory K. Tippin, Vikas Parihar, Joshua A. Rash

**Affiliations:** a Department of Psychology, Memorial University of Newfoundland, St. John’s, Newfoundland, Canada; b Michael G. DeGroote Pain Clinic, Hamilton Health Sciences, Hamilton, Ontario, Canada; c Department of Psychiatry & Behavioural Neurosciences, McMaster University, Hamilton, Ontario, Canada

**Keywords:** Cannabinoids, medical cannabis, chronic pain, Veterans, military, qualitative research

## Abstract

**Background:**

During fiscal year 2021–2022, Veterans Affairs Canada (VAC) reimbursed 18,388 veterans for medicinal cannabis at a cost of $153 million. Yet, it is not known whether the reimbursement program is producing a net benefit for veterans.

**Aims:**

This study investigated the views and experiences Canadian that veterans who live with pain have about medicinal cannabis use, including its use for the management of chronic pain, poor sleep, and emotional distress.

**Methods:**

Twelve Canadian veterans who live with pain—eight men, four women; split across four focus groups—were recruited to participate in a semistructured discussion around their experiences with medicinal cannabis use.

**Results:**

Using inductive thematic analysis, seven broad categories were identified: (1) cannabis use behaviors, (2) reasons for cannabis use, (3) outcomes from cannabis use, (4) facilitators of cannabis use, (5) barriers to cannabis use, (6) stigma around cannabis use, and (7) questions and concerns about cannabis use.

**Conclusions:**

Most veterans initiated cannabis use to manage the symptoms of preexisting medical and/or mental health conditions. Despite some negative side effects, most veterans reported improvements in their overall quality of life, sleep, relationships, mood, and pain. Concern remains around the discrepancy between veterans’ qualitative reports of beneficial outcomes from medicinal cannabis use and equivocal findings around the benefit-to-harm ratio in the wider literature. Currently, the VAC reimbursement program remains challenged by unclear indication for which veterans, with what condition(s), at what dose, and in what form medical cannabis is most beneficial.

## RÉSUMÉ

**Contexte:** Au cours de l’exercice 2021–2022, Anciens Combattants Canada (ACC) a remboursé 18 388 anciens combattants pour le cannabis médicinal, pour un coût de 153 millions de dollars. Pourtant, on ne sait pas si le programme de remboursement donne lieu à un bénéfice net pour les anciens combattants.

**Objectifs:** Cette étude porte sur les points de vue et les expériences des anciens combattants canadiens qui vivent avec la douleur sur la consommation de cannabis médicinal, y compris son utilisation pour la gestion de la douleur chronique, les problèmes de sommeil et la détresse émotionnelle.

**Méthodes:** Douze anciens combattants canadiens qui vivent avec la douleur - huit hommes et quatre femmes répartis en quatre groupes de discussion - ont été recrutés pour participer à une discussion semi-structurée autour de leurs expériences avec la consommation de cannabis médicinal.

**Résultats:** Une analyse thématique inductive a permis d’établir sept grandes catégories : (1) les comportements de consommation de cannabis, (2) les raisons de la consommation de cannabis, (3) résultats de la consommation de cannabis, (4) les facteurs qui facilitent la consommation de cannabis, (5) les obstacles à la consommation de cannabis, (6) la stigmatisation autour de la consommation de cannabis et (7) les questions et préoccupations concernant la consommation de cannabis.

**Conclusions:** La plupart des anciens combattants ont commencé à consommer du cannabis pour gérer les symptômes de maladies préexistantes et/ou des problèmes de santé mentale. Malgré certains effets secondaires négatifs, la plupart des anciens combattants ont signalé une amélioration de leur qualité de vie globale, de leur sommeil, de leurs relations, de leur humeur et de leur douleur. La préoccupation demeure autour de l’écart entre les rapports qualitatifs des anciens combattants décrivant les résultats bénéfiques de la consommation de cannabis médicinal et les résultats équivoques autour du rapport bénéfice/effet néfaste dans la littérature plus large. Actuellement, le programme de remboursement d’ACC reste contesté en raison d’indications peu claires concernant pour quels vétérans, atteints de quelles affections, à quelle dose et sous quelle forme le cannabis médical est le plus bénéfique.

## Introduction

Veterans experience greater incidence of medical conditions than the general population. Forty-one percent of Regular Force Canadian veterans report living with chronic pain or discomfort, which is about twice the rate in the Canadian general population.^1^ Canadian veterans with chronic pain are 11 times more likely to experience disabilities limiting their daily activity when compared to those without chronic pain.^2^ The presence of chronic pain is associated with a higher prevalence of comorbidities compared to those without chronic pain.^3^ For example, 79% of Canadian veterans with chronic pain report having a diagnosed mental health condition, and 83% report past-year suicidal ideation.^2,4^ Veterans also frequently report sleep-related difficulties associated with pain, such as insomnia, nightmares, and poor sleep quality.^5,6^ A systematic review of longitudinal studies found that a decline in sleep quality and quantity was associated with a two- to threefold increase in the risk of developing a pain condition and a decline in self-reported physical health status.^7^ A systematic scoping review arrived at a similar conclusion, reporting that treatment-induced sleep improvements enhanced chronic pain outcomes.^8^ Consistent with the comorbidity evident in the literature, Canadian veterans have indicated that optimizing chronic pain management as well as identifying and treating mental illness in veterans living with chronic pain are research priorities for their population.^9^

### Medicinal Cannabis in Canada

The use of cannabis for medical purposes among veterans has a long history in Canada. Formal reimbursement of medical cannabis by the Department of Veterans Affairs Canada (VAC) began in 2007. From 2008 to 2014, access to medical cannabis was limited by the Marihuana Medical Access Regulations, in which individuals required endorsement by a specialist physician to receive cannabis for medical use for select indications, including cancer, AIDS/HIV, multiple sclerosis, spinal cord injury or disease, epilepsy, or severe arthritis.^10^ The Marihuana for Medical Purposes Regulations replaced the Marihuana Medical Access Regulations in April 2014, allowing nonspecialist physicians to prescribe for patients and removing restrictions based on indication.^10^ Further revisions to legislation occurred in 2016 to allow authorized individuals to grow and purchase medical cannabis.

Medicinal cannabis regulatory changes occurring between 2014 and 2016 resulted in an increased number of veterans receiving medicinal cannabis authorization and reimbursement through VAC. Only 37 veterans were approved by VAC for medicinal cannabis authorizations in fiscal year 2011–2012 at a cost of $103,424.^11^ By fiscal year 2016–2017, the program had expanded, reimbursing 4474 veterans at a cost of $63,703,151.^11^ Due to the unforeseen increase in cannabis approvals among veterans, policy changes at VAC took place in 2016 to limit most recipients to 3 g of cannabis per day at a reimbursement rate of $8.50/g.^12^ With this policy change, costs decreased to $50,967,423 in fiscal year 2017–2018, despite an increase in the number of veterans reimbursed for cannabis of 2824 over the year before. The production, distribution, sale, and consumption of cannabis for recreational use became legal with the passing of the Cannabis Act by the Government of Canada in October 2018, leading to greater awareness and social acceptance of cannabis use.^13^ As of fiscal year 2021–2022, the number of veterans reimbursed has expanded to 18,388, with the total expenditure on cannabis exceeding $153 million.^11^ Despite the annual expenditure on cannabis, it is not known whether the VAC reimbursement program is producing an appreciable net benefit in the lives of the veterans involved, leading to the suggestion that reimbursement and oversight guidelines should possibly be revisited.^14^

### Veterans’ Perceptions of Medicinal Cannabis

Most research on the medicinal use of cannabis has been conducted on civilians and assessed the efficacy of synthetic derivatives of cannabis such as nabilone or dronabinol.^15–17^ The limited research that has been conducted on veteran populations was mostly conducted within the United States.^14,18–21^ In a qualitative cross-sectional case study on perceptions of cannabis use among 12 U.S. combat veterans, Croulet^19^ reported that all but one preferred cannabis over pharmaceutical medications for treating anxiety and sleep disturbances. In a qualitative study on cannabis risk perception in a sample of 31 U.S. veterans, Wilkinson et al.^18^ reported that most viewed cannabis as safe and not addictive and that this view was informed by individual experience with cannabis use. In an exploratory qualitative study of perceived risks and benefits of cannabis use with 23 early adult veterans and active-duty military members in the United States (aged 18–29; 10 veterans), Clary et al.^20^ reported that most perceived cannabis as an attractive alternative to opioid and benzodiazepine medications and an effective treatment for physical ailments and mental health issues. The authors also noted that veterans perceived cannabis as carrying limited risks. Further studies have reported that veterans view cannabis as an effective treatment for symptoms associated with posttraumatic stress disorder (PTSD),^22^ chronic pain,^23^ sleep disturbances,^24^ and emotional dysregulation.^25^ Though there is currently a lack of scientific consensus on the therapeutic benefit of cannabis for veterans, there is also little knowledge of Canadian veterans’ subjective experience of using cannabis medicinally.

### Aims

Little is known on the use of medicinal cannabis for the treatment of chronic pain, poor sleep, and symptoms of emotional distress among Canadian veterans living with pain. In particular, it is important to determine whether the use of medicinal cannabis has a positive, negative, or equivocal effect on the seven domains of veteran well-being as defined by the VAC, which include health, purpose, finances, social integration, life skills, housing and physical environment, and culture and social environment.^26^

The primary objective of this qualitative study was to develop a better understanding of the views and experiences that Canadian veterans who live with pain have about medicinal cannabis use, including its use for the management of chronic pain, poor sleep, and emotional distress.

## Methods

The goal of this qualitative study was to conduct four focus groups. Based on work by Guest et al.^27^ data saturation of 90% can usually be achieved with three to six focus groups, and most new information in a qualitative data set is generated early in the process, with a relatively sharp decline in new information occurring after just a small number of data collection/analysis events.^28^

### Participants

Participants were recruited through the Chronic Pain Center of Excellence for Canadian veterans. The Chronic Pain Center of Excellence has established a list of veterans who have consented to participate in research. A fan-out recruitment message detailing the goals of the focus group was sent to all 1135 veterans on the list at the time of recruitment. There is a significant gender imbalance in the Canadian Armed Forces (CAF), where one in six CAF members are women.^29^ We purposefully recruited women to reduce this gender imbalance and ensure that one-third of participants were women. Participation was voluntary, and interested veterans signed up and completed informed consent forms online. Participants were offered a $25 gift card for their time.

To be included in the study, a participant had to be a Canadian veteran—at least 18 years of age—with self-identified chronic pain who has or is currently using licensed medicinal cannabis authorized by the VAC. Individuals were excluded if they were nonveterans (i.e., active armed forces service members or civilians), were unable to read and write in English, were currently pregnant or breastfeeding, or were planning to become pregnant during the observation period.

### Procedure

This study was approved by the Hamilton Integrated Research Ethics Board. Focus groups were conducted using the Zoom virtual conferencing platform. Participants were sent guidelines to follow to ensure privacy and confidentiality during the focus group, such as using a private space and ensuring that others could not overhear the conversation or view participants.

Focus groups were recorded, transcribed verbatim using the NVivo^30^ transcription service, and de-identified. The video recordings were combined with their associated transcripts to identify the contributions of each participant who were assigned a unique participant identification number. Transcripts were not returned to participants for comment or correction.

### Focus Groups

Focus groups were conducted by members of the Healthcare Human Factors team, University Health Network, Toronto, and observed by G.T., V.P., and J.A.. The Healthcare Human Factors team was recruited owing to their expertise in facilitating focus groups to identify stakeholder priorities. Focus groups consisted of two to four veterans, lasted on average 90 min, and were conducted between February 16, 2022, and March 23, 2022. Men and women were in separate focus groups because of the high prevalence of sexual assault endured by women in the CAF at the hands of male service members.^31^ The women participating appreciated the opportunity to share their experiences in the exclusive company of other women. The research team developed a qualitative focus group guide that queried experiences with medicinal cannabis use (see Appendix). After introductions, participants were asked to reflect on a series of opening questions and then share their thoughts with the group one by one. Afterward, more specific questions were asked to uncover the research questions that veterans were most interested in getting answers to. The facilitators then reflected on the themes that had arisen and probed deeper to fill in gaps and flesh out thematic areas where necessary. For example, barriers and facilitators of medicinal cannabis use emerged as general themes and were probed more deeply.

### Data Synthesis

Transcripts were independently reviewed by D.S. and N.K.-V. to determine initial codes using open coding.^32^ A codebook with definitions of codes was derived through an iterative process of discussion and consensus. Discrepancies that could not be resolved through consensus were brought forward to the remaining authors for discussion and resolution. In accordance with grounded theory, inductive thematic analysis was used to identify common themes, and axial coding was used with each coder independently completing one of the two remaining focus group discussions.^32–34^ Individual quotations were linked to multiple codes as appropriate. The constant comparative method was used throughout this process, with codes and themes being continuously revisited to ensure that they fit the data.^33,34^ To be included, a subtheme had to have a combined value of ≥5, based on the number of focus groups in which the subtheme was raised plus the number of participants who endorsed that subtheme. The codebook was reviewed by all members of the team.

### Reflexivity

This review was undertaken by a multidisciplinary team with diverse viewpoints and experiences with the medicinal use of cannabis among veterans, including three practicing clinical psychologists (all male) with expertise in addictions and chronic pain management; one practicing pharmacist (male) with expertise in medicinal cannabis and chronic pain management; one research coordinator (female) with a background in medical science and experience with project management; one research assistant (female) pursuing a degree in applied psychological science with a background in qualitative and quantitative research, program evaluation, mental health and addictions, cannabis use, and chronic pain; and one trainee (male) pursuing a degree in clinical psychology with a background in experimental psychology, experience as a former research assistant with the Cannabis Health Evaluation Research Partnership (CHERP–MUN School of Pharmacy), and lived experience as a Canadian Armed Forces Veteran with 14 years of service in the reserve and regular forces combined. Members of our team engage in regular clinical practice and have clinical experience working with people—including veterans—who use medicinal cannabis to manage symptoms associated with chronic pain, poor sleep, and PTSD. Members of our team are also involved in federal mentorship programs for chronic pain, substance use, mental health, and addictions.

It is important that we acknowledge the lens through which our study team sought, coded, and identified emergent themes to enhance trustworthiness of findings. We have wide familiarity with chronic pain management, opioid use, medicinal cannabis, and veteran mental health in the fields of psychology, pharmacy, and medicine. Moreover, we have a keen interest in ensuring that the symptom management needs of veterans are being met in a safe, efficient, and effective manner. We strive to remain as objective as possible, focusing on what was explicitly discussed by the focus group participants, rather than trying to infer meaning during the coding process. We had a civilian (N.K.-V.) and a veteran (D.S.) conduct the initial coding and inductive thematic analysis independently to control for potential bias stemming from civilian and veteran perspectives.

## Findings

The final sample consisted of 12 Canadian veterans split across four focus groups. None of the veterans who consented to participate dropped out.

Demographic characteristics for the veterans who participated in this study are displayed in Table 1. Compared to the broader veteran population in Canada, our sample included a higher percentage of women (33% compared to 13%), veterans aged 20 to 39 (33% compared to 10%), and veterans aged 40 to 59 (50% compared to 29%) and a lower percentage of veterans aged 60+ (17% compared to 46%).^35^

**Table 1.** Veterans’ demographic characteristics.

Demographic characteristics	Participants	%
Gender		
Woman	4	33
Man	8	67
Race		
White	12	100
Age		
20 to 39	4	33
40 to 59	6	50
60+	2	17
Years served in the Canadian Armed Forces^a^		
<20 years	3	25
≥20 years	6	50
Element^b^		
Army	5	42
Navy	2	17
Air Force	4	33
Trade^c^		
Combat arms	2	17
Support	10	83
	Range	Mode
Years using cannabis medicinally^d^	1–10	4

*N* = 12, across four focus groups; focus groups 1 and 2 consisted of 4 men each, and focus groups 3 and 4 consisted of 2 women each.

^a^Three veterans did not report the number of years served.

^b^One veteran belonged to a purple trade (i.e., a trade not tied to a specific element) and did not specify the assigned element.

^c^Belonging to a combat arms trade is no guarantee that a soldier saw combat, nor is belonging to a support trade a guarantee that they did not. For example, a supply tech (a support trade) recounted experiences of coming under enemy fire in Afghanistan during supply runs.

^d^Two veterans did not report the precise number of years they have been using cannabis medicinally.

We identified 42 subthemes subsumed within 17 distinct themes and split across seven broader categories, including cannabis use behaviors, reasons for cannabis use, outcomes from cannabis use, facilitators of cannabis use, barriers to cannabis use, stigma around cannabis use, and questions and concerns about cannabis use. The emergent themes based on thematic analysis are displayed in Table 2.

**Table 2.** Emergent themes.

Theme
Cannabis use behaviors
1. First time using cannabis is reported to have occurred outside of the military, either before or after military service.
2. Typical duration of cannabis use for veterans is reported to be 4 years.
3. Most veterans report using ingestible forms of cannabis.
Reasons for cannabis use
4. Veterans report using cannabis to manage symptoms of preexisting—often comorbid—medical conditions, chronic pain foremost among them.
5. Veterans report initiating cannabis use to avoid undesirable side effects from prescription opioids and other pharmacological treatments for comorbid chronic pain and mental health concerns.
Outcomes from cannabis use
6. Veterans report being able to replace a number of pharmacological treatments with cannabis, which has led to improvements in their overall quality of life, sleep, relationships, and mood and a reduction in their symptoms.
7. Veterans report experiencing a number of negative side effects from using cannabis, as well as disliking the taste or smell of cannabis oils.
Facilitators of cannabis use
8. Veterans report that the VAC financially supported their request for medicinal cannabis.
9. Veterans report receiving support for obtaining medicinal cannabis from private medical cannabis clinics and the public system.
10. Veterans report receiving most of their information about cannabis from peers.
Barriers to cannabis use
11. Veterans report difficulties locating a family doctor, accessing health care services, and obtaining a stable supply of medicinal cannabis due to supply chain issues, as well as delays in access as a result of switching providers.
12. Veterans report challenges around cannabis travel legislation that limit their travel or restrict their ability to drive.
13. Veterans report lack of support/knowledge within the civilian health care system around cannabis use.
14. Veterans report unclear packaging and product information, as well as difficulty opening the packaging that their medicinal cannabis arrives in.
Stigma around cannabis use
15. Veterans report experiences of systemic stigma in the public health care system, public stigma, and self-stigma.
Questions and concerns about cannabis use
16. Veterans report having questions and concerns about the lack of clarity surrounding proper dosage and how to go about finding the correct dosage to treat specific symptoms without resorting to a trial-and-error approach.
17. Veterans report having questions about the long-term effects of cannabis use and the different terpene profiles found in cannabis.

A detailed subtheme breakdown is displayed in Table 3. Participants did not provide feedback on the findings.

**Table 3.** Summary of findings.

Subthemes	Subtheme description	Most representative quote	Count^a^
**Cannabis use behaviors**			
Theme 1: First time using cannabis is reported to have occurred outside of the military, either before or after military service.			
1.1 First time using cannabis	Veterans report using cannabis for the first time outside of the military, with some using before entering the military (*N* = 3, *n =* 4) and others first using after leaving the military (*N* = 2, *n =* 3).	“I used a lot of cannabis when I was younger before I joined, completely stopped while I served, and I restarted maybe a month after release.” (F2, V6)	*N =* 3, *n* = 4
		“I got it 2013 or 2014, so it was a long time after I left the military, because we didn’t talk about pot in the military.” (F4, V11)	*N* = 2, *n* = 3
Theme 2: Typical duration of cannabis use for veterans is reported to be 4 years.			
2.1 Duration of current use	Veterans report using cannabis for the past 3 to 7 years (mode = 4 years)	“It’s been about four years now.” (F1, V2)	*N* = 2, *n* = 6
Theme 3: Most veterans report using ingestible forms of cannabis.			
3.1 Capsules/pills	Veterans report ingesting cannabis capsules/pills.	“I’m taking CBD gel caps.” (F3, V10)	*N* = 4, *n* = 5
3.2 Edibles	Veterans report ingesting cannabis edibles.	“I started with the plant smoking and I found that my throat was aggravated by that throat and sinus is so I switched to vaping and that didn’t help much either so I switched to edibles.” (F1, V3)	*N =* 2, *n* = 3
**Reasons for cannabis use**			
Theme 4: Veterans report using cannabis to manage symptoms of preexisting—often comorbid—medical conditions, chronic pain foremost among them.			
4.1 Chronic pain	Veterans report using cannabis to manage symptoms of chronic pain.	“I find it useful, it controls the pain for me.” (F1, V1)	*N* = 4, *n* = 8
4.2 Comorbid conditions	Veterans report using cannabis to manage symptoms from multiple medical conditions.	“My whole body is pretty much just broken from head to toe, same as Veteran 3, you know, crawling under aircraft lift and everything, the whole bit. So I’ve got bad knees, bad feet, bad back, neck, migraines, depression, and that was what got me into the cannabis.” (F1, V2)	*N* = 3, *n* = 7
4.3 Musculoskeletal injury	Veterans report using cannabis to manage symptoms resulting from a musculoskeletal injury.	“The legalization of cannabis came at probably a perfect time for me because I was dealing with all the back issues.” (F1, V3)	*N* = 3, *n* = 6
4.4 Sleep disturbances	Veterans report using cannabis to manage sleep disturbances and/or improve sleep quality.	“Smoking more or less helps me sleep.” (F1, V4) “The THC for me helps me sleep.” (F3, V9)	*N* = 2, *n* = 5
4.5 PTSD	Veterans report using cannabis to manage symptoms of PTSD.	“So with that medical marijuana definitely has impacted me greatly in my progress in dealing with PTSD symptoms, symptom treatment for mental health. […] So this stuff is incredibly effective.” (F2, V7)	*N* = 3, *n* = 4
4.6 Depression	Veterans report using cannabis to manage symptoms of depression.	“Five years ago I was suffering from depression which I didn’t know it at the time ’cause I was always angry pushing my family and people away from me stand in my basement and stuff and it was through my psychologist that she—she suggested it because the antidepressant meds just weren’t working.” (F1, V2)	*N* = 1, *n* = 4
4.7 Anxiety	Veterans report using cannabis to manage symptoms of anxiety.	“I mostly use CBD for either anxiety, relaxation throughout the day.” (F2, V6)	*N* = 2, *n* = 3
Theme 5: Veterans report initiating cannabis use to avoid undesirable side effects from prescription opioids and other pharmacological treatments for comorbid chronic pain and mental health concerns.			
5.1 Undesirable side effects from prescription opioids	Veterans report negative side effects resulting from prescription opioid use such as stomach pain, endocrine dysregulation, flat affect, suicidality, and addiction.	“Just like Veteran 11, I cannot do those drugs for pain, it just killed my stomach. So between having pain in my stomach and pain in my joints and everything else, I’m just going to choose the pain in my joints because it’s … at least I know why I have it.” (F4, V12)	*N* = 3, *n* = 7
5.2 Undesirable side effects from Gabapentin	Veterans report negative experiences with or effects from using gabapentin for pain management such as suicidality, mental fog, and exacerbation of PTSD symptoms.	“The doctor put me on a drug called gabapentin. It has side effects so I suffered through the minor side effects but I never really read the instructions on it fully and one of the severe side effects is suicidal thoughts … one day driving along I just started driving toward a big truck coming toward me, so that made me realize something is not correct here … went and saw a therapist and she said, ‘This is your problem. That medication.’ … But the problem with the gabapentin made me realize like this isn’t the way to go. So I said to my doctor, … “What about this cannabis stuff? Would that help me?” (F2, V8)	*N* = 3, *n* = 3
**Outcomes from cannabis use**			
Theme 6: Veterans report being able to replace a number of pharmacological treatments with cannabis, which has led to improvements in their overall quality of life, sleep, relationships, and mood and a reduction in their symptoms.			
6.1 Replaced other pharmacological treatments	Veterans report that they were able to replace multiple pharmacological treatments with cannabis.	“I feel good on this. I have stopped over-the-counter meds, have stopped other medications, so I’m just strictly right now on cannabis. I think that’s good.” (F2, V8)	*N* = 3, *n* = 10
6.2 Improved overall quality of life	Veterans report using cannabis has improved their overall quality of life.	“When I’m coming along good and I’m walking, I’m out with the dog and doing my yard work and having … the best day of my life, I’m really happy that the CBD is working its magic.” (F4, V11)	*N* = 3, *n* = 7
6.3 Improved sleep	Veterans report that using cannabis has improved their sleep quality and/or duration.	“I use a bit of THC at night because it’s a bit better for the nightmare portion.” (F2, V6)	*N* = 4, *n* = 6
6.4 Improved relationships	Veterans report that using cannabis has had a positive impact on relationships with friends and family.	“One thing I’ve found using cannabis is getting my life back, as I’m actually now able to enjoy my grandkids. I’m able to get on the floor play with them and stuff.” (F1, V2)	*N* = 2, *n* = 5
6.5 Improved mood	Veterans report using cannabis has improved their mood.	“For my mood, my anxiety, it really helps down. Like, that’s going to calm me down.” (F4, V12)	*N* = 3, *n* = 4
6.6 Managed symptoms effectively	Veterans report perceiving cannabis to be an effective medication for the management of their symptoms of chronic pain, PTSD, and tremors.	“I have tremors, oil is going all over the place. So I found CBD capsules. So I started taking them, they’re nice and easy and very helpful. I found the high doses of CBD in them help in calming down the tremors I used to have—bolts of lightning shoot down into my hands my feet and all of that kind of stuff. Ninety percent gone.” (F2, V8)	*N* = 3, *n* = 3
Theme 7: Veterans report experiencing a number of negative side effects from using cannabis, as well as disliking the taste or smell of cannabis oils.			
7.1 Unpleasant taste or smell specific of cannabis oils	Veterans report that they found the smell or taste of cannabis oils to be unpleasant or unpalatable.	“I started with oil and the taste and the smell was disgusting, so I went with gel caps and it’s basically all I use right now.” (F1, V2)	*N* = 3, *n* = 4
7.2 Anxiety, paranoia, and intoxication associated with high cannabis dosage	Veterans report that taking too high a dose of cannabis made them feel intoxicated, anxious, and/or paranoid.	“I get paranoid if I take too much, so I will get paranoid really easy, so that’s always finding that little perfect sweet spots. But, yeah, it’s usually decent.” (F2, V6)	*N* = 3, *n* = 4
7.3 General side effects associated with cannabis-use irrespective of dosage	Veterans report that they may experience dry mouth, throat/sinus irritation, increased appetite, and weight gain when using cannabis.	“For me, it’s weight gain. I get the munchies really bad, ’specially in the evening if I’ve been on the cannabis all day, I–my weight has gone up I guess a good 25 to 30 pounds.” (F1, V1)	*N* = 2, *n* = 4
**Facilitators of cannabis use**			
Theme 8: Veterans report that VAC financially supported their request for medicinal cannabis.			
8.1 VAC coverage	Veterans report obtaining financial coverage for medical cannabis with relative ease through VAC.	“Weirdly enough, getting coverage was super easy. It’s three months of struggle to get my driveway clean, but $1000 bucks of cannabis a month was not even hard. I find it weird, but who am I to judge?” (F2, V6)	*N* = 4, *n* = 5
Theme 9: Veterans report receiving support for obtaining medicinal cannabis from private medical cannabis clinics and the public system.			
9.1 Private medical cannabis clinics for veterans	Veterans report their experiences with private medical cannabis clinics, which specifically serve the needs of veterans, facilitating prescriptions, reimbursement, scheduling, and the coordination of health care, as well as providing information, social activities, and peer support for veterans.	“I went through CannaConnect here in Kingston just because they helped with all the paperwork, getting the doctor, you know, getting the prescription with Veterans Affairs and the whole bit right, so I didn’t really have to do anything other than show up for doctors’ appointments and still with [CannaConnect] if I have questions or anything you know that there’s a point of contact. I contact her and she’s really quick to respond, give me the answers I need, that sort of thing, and she’s been really helpful.” (F1, V2)	*N* = 3, *n* = 7
9.2 Public system	Veterans experience support from the public systems mostly reported in mental health care providers including psychiatrists and psychologists (*N =*4, *n* = 4); some also report support from health care professionals including physicians and nursing committees (*N* =1, *n* = 2).	“[reading prompt] So who recommended or prescribed it? I believe it was my psychiatrist originally. I was still in … because of my neurological problems, and then also because I was diagnosed with PTSD, severe PTSD. He initially prescribed. …” (F3, V10)	*N* = 4, *n* = 4
		“My family doctor was quite prevalent in getting me involved with the medical marijuana.” (F1, V1)	*N* = 1, *n* = 2
Theme 10: Veterans report receiving most of their information about cannabis from peers.			
10.1 Peers	Veterans report that their primary source of information on cannabis is from peers, family, and/or friends.	“I still have my friends that I see, they’re all using cannabis now, too, so we’re all in the same boat, we’re all getting older, or pain is starting to develop more. We’re all ex-military and sort of been used hard and put away wet and we didn’t get the proper brush down we should have been getting. [I imagine you probably learn a little bit from each other’s experiences using cannabis is that fair to say.] “Oh, yes, yeah, we play on each other’s past the benefits and downfalls as well. I know the edibles really don’t […] work too well for me, but the capsules and the oil do and I’ve got friends where it’s the opposite way, […] so we just use each other.” (F1, V1)	*N* = 2, *n* = 4
**Barriers to cannabis use**			
Theme 11: Veterans report difficulties locating a family doctor, accessing health care services, and obtaining a stable supply of medicinal cannabis due to supply chain issues, as well as delays in access as a result of switching providers.			
11.1 Difficulty accessing health care	Veterans report experiencing difficulty finding a family doctor or accessing health care services.	“Trying to find a family doctor is next to impossible, they’re totally busy and doctors here right now, we’re actually doing telephone calls, so there’s no office work anymore.” (F1, V1)	*N* = 4, *n* = 8
11.2 Supply issues	Veterans report difficulties obtaining a stable supply of cannabis.	“I have a hard time filling [prescriptions] because the only provider … originally my provider was MedReleaf but then MedReleaf got swallowed by the mother company of Aurora, which is out of BC. … I don’t know if all of a sudden CBD has become so popular that’s why they’re never in stock for the stuff that I usually order.” (F3, V9)	*N* = 2, *n* = 4
11.3 Difficulty switching providers	Veterans report issues in access due to the difficulties involved in switching their provider of medical cannabis.	“You can switch providers but there is a delay to it and it messes with your order sequence or order timings, I guess, because when you switch from one provider to another provider, your order date then changes and potentially the product you’re ordering can change too, so if one provider doesn’t have what you had before, you’re trying to figure out what’s going to work for you again and then you could go through 2–3 months of trial and experimentation to figure out what’s going to work for you on a new provider.” (F1, V3)	*N* = 2, *n* = 4
Theme 12: Veterans report challenges around cannabis travel legislation that limit their travel or restrict their ability to drive.			
12.1 Limits travel	Veterans report cannabis medication limiting or restricting their ability to travel between provinces or internationally.	“Travel is a big negative thing, too, because … we want to sell our place here in Canada and start traveling … and that’s … kind of got me worried because I don’t want to go back on opioids.” (F1, V3)	*N* = 3, *n* = 6
12.2 Restricts driving	Veterans report cannabis medication restricting their driving.	“The other problem is driving with this 12 hours bottle a throttle type of thing. I know when I was on Percocet the rules are totally different than if you’re on cannabis since they’ve relaxed the laws for the cannabis use now with the medication or the dosage that I’m not–it’s 12 hour bottle to throttle type of thing and you have to plan your trips, you have to know almost in advance which you’re gonna do, either that or you have to cancel your trip altogether.” (F1, V1)	*N* = 3, *n* = 3
Theme 13: Veterans report lack of support/knowledge within the civilian health care system around cannabis use.			
13.1 Lack of scientific medical knowledge around cannabis	Veterans report perceived lack of medical and/or scientific knowledge around cannabis and its proper use.	“Went back to my family doctor, said, ‘What about this cannabis stuff, would that help me?’ And she kind of looked at me like a deer in the headlights, like, she didn’t know about cannabis and medical uses. She had heard about it, but she didn’t know the details.” (F2, V8)	*N* = 4, *n* = 5
13.2 Lack of guidance	Veterans report the perception that there is a lack of guidance from medical professionals and suppliers on how to use cannabis and how much to take.	“[discussing lack of guidance on dosage when starting cannabis] I was pissed because nobody told me. Because I didn’t know. … I learned very quickly ‘no, no, no’ you take a little bit to start, like, I think, what they say 0.5 mL of the CBD. Do that for about a week, and then increase to the next 0.5 and then. Oh! Well! that would have been nice to know before I ended up stoned out of my tree. Thanks. ‘Oh, we didn’t tell you?’ No! You didn’t even give me booklets, pamphlets, or nothing when it started. And I didn’t get angry, I just more or less laughed it off ’cause, you know, what do you do?” (F3, V9)	*N* = 3, *n* = 4
13.3 Overreliance on narcotics for pain	Veterans report experiences of being prescribed far more opioids to manage their pain than they felt they needed and that this was done without giving due consideration to alternatives such as cannabis.	“When I fractured my spine, so I fractured my L4 and L5 vertebrae, they immediately went to oxy’s [i.e., Oxycodone]. They said, ‘Here’s a 30 count of oxy, take it. It’s going to make you feel better.’ … I looked at it, I’m like, ‘Should I actually take this or not?’ Fifteen days go by. I go back to another appointment, they’re like, ‘Oh, so did you take did you take the medication?’ I lied to them said, ‘Yes, I did.’ They gave me another 30 count of boxes. It’s just like, like, what the hell are they doing? You can get addicted after taking 30 of those pills, and they gave me 60 in 30 days. I didn’t take them, but, like, this is how preposterous it is.” (F2, V5)	*N* = 2, *n* = 5
Theme 14: Veterans report unclear packaging and product information, as well as difficulty opening the packaging that their medicinal cannabis arrives in.			
14.1 Product information and packaging	Veterans report confusion due to unclear product information, poor packaging, and difficulty opening the bottles in which their medicinal cannabis is packaged.	“They take me 5 minutes to figure out how to open them, there are more like a kid’s game. It’s like, this is some business and new kid’s game so, yeah, it’s something, when it comes to packaging we need to—it’s they just applied the same packaging we do with everything else.” (F1, V4)	*N* = 2, *n* = 3
**Stigma around cannabis use**			
Theme 15: Veterans report experiences of systemic stigma in the public health care system, public stigma, and self-stigma.			
15.1 Stigma within the public health care system	Veterans report stigmatizing experiences within the public health care sector around disclosing cannabis use or requesting a medicinal cannabis prescription (e.g., being dismissed as an addict).	“[During] the intake for my nose surgery when I … went through the list of drugs, cannabis, of course, wasn’t a drug anymore, it’s legal, but then when we got to that point where we had to discuss my cannabis use it went from I was a normal person to the look on her face was—she was … suddenly looking at an addict, and I could feel the temperature in the room change. It was bizarre.” (F1, V4)	*N* = 2, *n* = 3
15.2 General experiences of stigma around cannabis use within society	Veterans report general experiences of stigma against cannabis within society largely stemming from a prelegalization “War on Drugs”–era mindset.	“I’ll use the example of … my mother-in-law [who] is against cannabis: it’s a drug. [She has a] little older mentality; it’s been illegal [her] whole life. They still see it as a drug. … At one point I walked out of the base hospital with a patch of fentanyl [on] my shoulder, hydromorphone by mouth, and even Vicodin as a breakthrough. So I had three hardcore narcotics that can easily kill you, especially the three together, but she saw it [as] better than using CBD.” (F2, V6)	*N* = 3, *n* = 7
15.3 Self-stigma (i.e., the internalization of systemic and public stigma around cannabis use) has had a divergent impact on decision to use cannabis	There was diverging evidence about the impact of self-stigma on the decision to use cannabis, with some veterans reporting an impact on their decision to use (*N =* 3, *n* = 5) and others reporting no impact (*N* = 3, *n* = 4).	“[Stigma] impacted my start with [cannabis]. I wish I’d started, you know, a couple of years before I actually did, but that was all delayed because of stigma.” (F1, V3)	*N* = 3, *n* = 5
		“[Reading prompt] Do others’ perceptions of cannabis impact your cannabis use? No, I could care less what other people think about it because I’m not taking a long list of drugs anymore and it helps me substantially.” (F3, V10)	*N* = 3, *n* = 4
**Questions and concerns stemming from a lack of resources to obtain accurate information on cannabis use and potential risk**			
Theme 16: Veterans report having questions and concerns about the lack of clarity surrounding proper dosage and how to go about finding the correct dosage to treat specific symptoms without resorting to a trial-and-error approach.			
16.1 Challenges surrounding proper dosage	Veterans are concerned by a lack of clarity surrounding proper dosage, difficulty finding the right dosage, and/or having to use trial-and-error to find the correct dosage.	“I think I had some ups and downs with it to get the dosage right, but now that it is [right] it seems to be working fairly well. Not as good as I wanted it to, but I think it’s just a matter of tweaking other things too.” (F1, V2)	*N* = 3, *n* = 6
16.2 Treating specific symptoms	Veterans report wanting more information about how to use cannabis to more effectively manage specific symptoms.	“That’s the place I would like to be, to see people getting more educated about what is the actual use of cannabis for somebody with PTSD, nightmare, et cetera, compared to ‘OK, so instead of using CBD, we’re going to give you a narcotic for the pain, we’re going to give you an antipsychotic, we’re going to give you this, going to give you this.’” (F2, V6)	*N* = 3, *n* = 3
Theme 17: Veterans report having questions about the long-term effects of cannabis use and the different terpene profiles found in cannabis.			
17.1 Long-term effects	Veterans report questions and concerns they have about the long-term effects of using cannabis.	“I think the biggest thing that I struggle with is what’s the daily marijuana use going to do to my mind in 30 years? […] I haven’t really been able to see anything or any type of studies or reports actually saying what prolonged marijuana use does to someone’s brain. I don’t know if it’s all just a wives’ tale. I don’t know if they said that to deter people from using marijuana [in the past], right? I think that for me … that’s my biggest question. … Everything else I’m pretty good with, but I think it’s just the long-term impacts of using it [that] I’m interested in.” (F2, V5)	*N* = 2, *n* = 6
17.2 Terpene profiles	Veterans report wanting to know more about the different terpenes within cannabis.	“A user’s guide for the terpenes to understand how each one affects the body, because they all have different—they all have different effects.” (F1, V4)	*N* = 2, *n* = 3

^a^*N* = number of focus groups in which subtheme was endorsed; *n* = number of veterans who endorsed subtheme.

### Cannabis Use Behaviors

Timing of cannabis initiation was the first theme that emerged, with initiation occurring either prior to joining the military (*n* = 4, 33%) or after release from military service (*n* = 3, 25%):

I used a lot of cannabis when I was younger before I joined, completely stopped while I served, and I restarted maybe a month after release. (Male veteran)

Most veterans reported having used cannabis for the past 3 to 7 years with a mode of 4 years. The most common form of medicinal cannabis used was ingestible, with some veterans taking cannabis capsules or pills (*n* = 5, 42%) and others taking edibles (*n* = 3, 25%).

### Reasons for Cannabis Use

The most cited reason for using cannabis was to manage the symptoms of preexisting conditions, with comorbid conditions (e.g., pain, depression, anxiety) being the norm (*n* = 7, 58%):

My whole body is pretty much just broken from head to toe, same as Veteran 3, you know, crawling under aircraft lift and everything, the whole bit. So, I’ve got bad knees, bad feet, bad back, neck, migraines, depression, and that was what got me into cannabis. (Male veteran)

Regarding specific medical conditions, veterans endorsed chronic pain (*n* = 8, 67%), musculoskeletal injury (*n* = 6, 50%), sleep disturbances (*n* = 5, 42%), PTSD (*n* = 4, 33%), depression (*n* = 4, 33%), and anxiety (*n* = 3, 25%). One of the primary motivators for veterans switching from other pharmacological treatments for their preexisting medical conditions to medicinal cannabis was avoidance of undesirable side effects. Many veterans expressed that negative side effects from taking prescription opioids (e.g., stomach pain, endocrine dysregulation, flat affect, suicidality, addiction) motivated their switch to cannabis (*n* = 7, 58%):

Just like Veteran 11, I cannot do those drugs for pain, it just killed my stomach. So, between having pain in my stomach and pain in my joints and everything else I’m just going to choose the pain in my joints because it’s … at least I know why I have it. (Female veteran)

Several veterans (*n* = 3, 25%) also reported undesirable effects from taking gabapentin (e.g., suicidality, mental fog, exacerbation of symptoms of PTSD) as a reason for switching to cannabis.

### Outcomes from Cannabis Use

Veterans reported that being able to replace multiple pharmacological treatments with cannabis had resulted in beneficial outcomes (*n* = 10, 83%):

I feel good on [cannabis]. I have stopped over the counter meds, have stopped other medications, so I’m just strictly right now on cannabis. I think that’s good. (Male veteran)

Specifically, veterans reported that being able to replace multiple pharmacological treatments with cannabis had improved their overall quality of life (*n* = 7, 58%):

When I’m coming along good and I’m walking, I’m out with my dog and doing my yard work and having … the best day of my life. I’m really happy that the CBD is working its magic. (Female veteran)

Veterans also reported that switching to cannabis improved their sleep (*n* = 6, 50%), relationships with friends and family (*n* = 5, 42%), and mood (*n* = 4, 33%) and allowed them to manage their symptoms more effectively (*n* = 3, 25%). Veterans did report potential negative side effects from cannabis use, such as an unpleasant taste or smell of cannabis oils (*n* = 4, 33%), and feeling intoxicated, anxious, and/or paranoid after using too much cannabis (*n* = 4, 33%):

I get paranoid if I take too much, so I will get paranoid really easy, so that’s always finding that little perfect sweet spot. But yeah, it’s usually decent. (Male veteran)

Some veterans reported general side effects of cannabis use irrespective of the dose consumed, such as dry mouth, throat or sinus irritation, increased appetite, and weight gain (*n* = 4, 33%).

### Facilitators of Cannabis Use

Veterans reported that receiving financial support for medicinal cannabis from the VAC was easy (*n* = 5, 42%):

Weirdly enough, getting coverage was super easy. It’s three months of struggle to get my driveway clean, but $1000 bucks of cannabis a month was not even hard. I find it weird, but who am I to judge? (Male veteran)

More than half of veterans interviewed reported receiving support for obtaining medicinal cannabis from private medical cannabis clinics (*n* = 7, 58%). These private medical cannabis clinics specifically serve the needs of veterans by filling prescriptions, facilitating reimbursement, providing suggestions for cannabis products to try, as well as scheduling and coordinating health care services. They also provide information, social activities, and peer support for veterans:

I went through [a private cannabis clinic] here in [a Canadian city] just because they helped with all the paperwork getting the doctor, you know, getting the prescription with Veterans Affairs and the whole bit, right. So, I didn’t really have to do anything other than show up for doctors’ appointments and still with [a private cannabis clinic] if I have questions or anything you know that there’s a point of contact. I contact her and she’s really quick to respond, give me the answers I need, that sort of thing and she’s been really helpful. (Male veteran)

Some veterans reported experiencing support from members of the public health care system for their cannabis use. For example, some veterans reported feeling supported in their decision to use medicinal cannabis from psychiatrists and psychologists (*n* = 4, 33%), as well as from nurses and family physicians (*n* = 2, 17%). Veterans reported that their primary source of information about cannabis is from peers, family, and/or friends (*n* = 4, 33%):

I still have my friends that I see they’re all using cannabis now too so we’re all in the same boat. We’re all getting older, or pain is starting to develop more. We’re all ex-military and sort of been used hard and put away wet and we didn’t get the proper brush down we should have been getting. … We play on each other’s past. … I know the edibles really don’t […] work too well for me but the capsules in the oil do and I’ve got friends where it’s the opposite way … so we just use each other. (Male veteran)

### Barriers to Cannabis Use

A majority of veterans reported that difficulty finding a family physician or accessing health care services was a barrier to medicinal cannabis use (*n* = 8, 67%). Some veterans reported difficulty obtaining a stable supply of medicinal cannabis (*n* = 4, 33%). Others noted disruptions in their access to medicinal cannabis due to difficulties in switching providers (*n* = 4, 33%):

You can switch providers but there is a delay to it and it messes with your order sequence or order timings I guess, because when you switch from one provider to another provider your order date then changes and potentially the product you’re ordering can change too so if one provider doesn’t have what you had before you’re trying to figure out what’s going to work for you again and then you could go through 2 to 3 months of trial and experimentation to figure out what’s going to work for you on a new provider. (Male veteran)

Restrictions placed on one’s ability to travel between provinces or internationally was a common complaint regarding cannabis medication (*n* = 6, 50%):

Travel is a big negative thing too because … we want to sell our place here in Canada and start traveling … and that’s … kind of got me worried because I don’t want to go back on opioids. (Male veteran)

Concern about cannabis-related driving restrictions was also noted (*n* = 3, 25%).

A lack of support and knowledge within the civilian health care system around medicinal cannabis use was another theme to emerge. Several veterans perceived that civilian health care providers had insufficient scientific medical knowledge around cannabis (*n* = 5, 42%):

Went back to my family doctor, said, “What about this cannabis stuff, would that help me?” And she kind of looked at me like a deer in the headlights, like she didn’t know about cannabis and medical uses. She had heard about it, but she didn’t know the details. (Male veteran)

Related to this, many veterans expressed insufficient guidance around how to use cannabis (e.g., quantity and timing of dosage) from medical professionals and cannabis suppliers (*n* = 4, 33%). Veterans reported an overreliance on prescribing narcotics for pain management by health care professionals. Many shared experiences where they felt that they were prescribed more opioids to manage their pain than was necessary and that this was done without giving due consideration to alternatives such as cannabis (*n* = 5, 42%):

When I fractured my spine … they immediately went to oxy’s [i.e., Oxycodone]. They said, “Here’s a 30 count of oxy, take it. It’s going to make you feel better.” … I looked at it, I’m like, “Should I actually take this or not?” Fifteen days go by. I go back to another appointment they’re like, “Oh so did you take the medication?” I lied to them, said, “Yes, I did.” They gave me another 30 count of boxes. It’s just like, like what the hell are they doing? You can get addicted after taking 30 of those pills, and they gave me 60 in 30 days. I didn’t take them, but like this is how preposterous it is. (Male veteran)

Several veterans also reported confusion due to unclear product information, poor packaging, and difficulty opening the bottles in which their medicinal cannabis was packaged (*n* = 3, 25%).

### Stigma around Cannabis Use

One in four veterans interviewed reported stigmatizing experiences within the public health care system around disclosing cannabis use or requesting a medicinal cannabis prescription (*n* = 3, 25%):

[During] the intake for my nose surgery when I … went through the list of drugs, cannabis of course wasn’t a drug anymore, it’s legal, but then when we got to that point where we had to discuss my cannabis use, it went from I was a normal person to the look on her face was, she was … suddenly looking at an addict and I could feel the temperature in the room change. It was bizarre. (Male veteran)

A majority of veterans reported experiences of stigma around cannabis use within society that largely stemmed from a prelegalization “War on Drugs”–era mindset (*n* = 7, 58%):

I’ll use the example of … my mother-in-law [who] is against cannabis: it’s a drug. [She has a] little older mentality; it’s been illegal [her] whole life. They still see it as a drug. … At one point I walked out of the base hospital with a patch of fentanyl [on] my shoulder, hydromorphone by mouth, and even Vicodin as a breakthrough. So, I had three hardcore narcotics that can easily kill you, especially the three together, but she saw it [as] better than using CBD. (Male veteran)

Self-stigma (i.e., the internalization of systemic and public stigma around cannabis use) had a divergent impact on veterans’ decision to use cannabis. Some veterans reported that self-stigma delayed their adoption of medicinal cannabis (*n* = 5, 42%):

[Stigma] impacted my start with [cannabis] I wish I’d started you know a couple of years before I actually did, but that was all delayed because of stigma. (Male veteran)

However, one in three veterans interviewed reported that systemic and public stigma did not impact their decision to use cannabis (*n* = 4, 33%):

I could care less what other people think about it because I’m not taking a long list of drugs anymore and it helps me substantially. (Female veteran)

### Questions and Concerns about Cannabis Use

Veterans reported a lack of clarity surrounding proper cannabis dosage and noted difficulty determining the correct dose to treat specific symptoms without resorting to a trial-and-error approach to find the correct dosage (*n* = 6, 50%):

I think I had some ups and downs with it to get the dosage right, but now that it is [right] it seems to be working fairly well. Not as good as I wanted it to, but I think it’s just a matter of tweaking other things too. (Male veteran)

Several veterans wanted more information about how to use cannabis to effectively manage specific symptoms (*n* = 3, 25%). For example, what specific strains, dosages, and schedules of cannabis consumption are advisable to manage symptoms of PTSD versus chronic pain? Veterans also had questions and concerns about the potential long-term effects of using cannabis (*n* = 6, 50%):

I think the biggest thing that I struggle with is what’s the daily marijuana use going to do to my mind in 30 years? … I haven’t really been able to see anything or any type of studies or reports actually saying what prolonged marijuana use does to someone’s brain. I don’t know if it’s all just a wives’ tale. I don’t know if they said that to deter people from using marijuana [in the past], right? I think that for me … that’s my biggest question. … Everything else I’m pretty good with, but I think it’s just the long-term impacts of using it [that] I’m interested in. (Male veteran)

There was also some interest in knowing more about the specific effects of the different terpenes—naturally occurring chemical compounds—within cannabis (*n* = 3. 25%).

### Themes Requiring Additional Exploration

There were 21 subthemes subsumed within 11 distinct themes that did not meet the threshold to be included in the summary of findings table. Almost all subthemes in this table were endorsed by only one or two veterans. Two subthemes were endorsed by three veterans but within a single focus group. Veterans were interested in learning more about how to transition from other medications to cannabis (*n* = 3, 25%), as well as what ratios of THC to CBD are optimal for the management of different symptoms (*n* = 3, 25%). Table 4 details the themes requiring additional exploration.

**Table 4.** Themes requiring additional exploration.

Subtheme	Subtheme description	Most representative quote	Count^a^
**Cannabis use behaviors**			
Theme 18: Some veterans report taking a moderate quantity of dry cannabis or oil in the morning and/or night, in some cases cannabis alongside other substances, and experimenting with different strains of cannabis.			
18.1 Dosage and timing	Veterans report taking 2–3 g of dry cannabis or 0.5 mL of cannabis oil per day, am and/or pm.	“It’s usually after supper around 7:30 and the biggest dose I take on the THC is half a mL is what I take, and that’s enough to help me sleep through the night.” (F3, V9)	*N* = 2, *n* = 2
18.2 Polysubstance use	Veterans report using cannabis alongside alcohol or prescription medications for sleep and pain.	“Meanwhile, I was drinking and smoking marijuana the whole time.” (F2, V5)	*N* = 2, *n* = 2
18.3 Cannabis strains used	Veterans report using indicas and sativas.	“I went on my voc rehab in September and since then I’ve played around with different sativas and things. I’ve been playing around with different strains and whatnot as well to try and figure out what works better than others.” (F1, V4)	*N* = 1, *n* = 2
Theme 19: Some veterans report using multiple forms of cannabis together, as well as using oils, smoking, nasal sprays, and creams.			
19.1 Multiple forms	Veterans report using multiple forms of cannabis concurrently.	“So it was basic medical marijuana. There was no fun stuff or anything like that, it was the drops and little vape pens and that was it. Oh, and tablets that had the pot inside it.” (F4, V11)	*N* = 2, *n* = 2
19.2 Oils	Veterans report using cannabis oils.	“My main go-to in the cannabis world is CBD oil.” (F3, V9)	*N* = 2, *n* = 2
19.3 Smoking	Veterans report smoking cannabis.	“I do smoke—I do smoke a fair bit of flower.” (F1, V4)	*N* = 1, *n* = 1
19.4 Nasal spray	Veterans report using cannabis nasal sprays.	“When I get migraines I’m able to manage it with—with a nasal spray meant for migraines, and with the THC it keeps it enough that I’m not going to be lying in my room with a pillow over my head for 8 hours.” (F1, V2)	*N* = 1, *n* = 1
19.5 Topical forms	Veterans report using topical cannabis products (e.g., creams).	“I and a lot of other veterans that I know get that go through Spartan Wellness is pain creams. … One is for muscles and one is for joints. … It really does help myself and many others.” (F3, V10)	*N* = 1, *n* = 1
**Reasons for cannabis use**			
Theme 20: Some veterans report the need for frequent dose adjustments of their opioid medications as a reason for switching to cannabis.			
20.1 Need for frequent dose adjustment of opioid medication	Veterans report that they had to frequently increase the dose of their opioid medication to manage their symptoms.	“The opioids are no longer working, so I go on higher and higher doses, and I was just becoming a BLOB on the couch.” (F1, V3)	*N* = 1, *n* = 2
Theme 21: Some veterans report negative experiences with selective serotonin reuptake inhibitors (SSRIs) and nabilone as reasons for switching to cannabis.			
21.1 SSRIs	Veterans report negative experiences with or effects from using SSRIs to manage symptoms of depression.	“The SSRIs pretty much turned me into a sociopath. I had almost no emotional—I’m registering with almost anything it would have to be almost through the roof for me to even sort of acknowledge that there should be an emotion reacted with this, whereas with cannabis, you know, what if it’s a sad movie, I’ll cry just straight up. I mean, that’s the biggest thing I’ve noticed is that I’m actually able to feel my emotions.” (F1, V4)	*N* = 1, *n* = 1
21.2 Nabilone (synthetic THC)	Veterans report negative experiences with or effects from using nabilone.	“Initially I was on the synthetic THC. […] Nabilone, I think. I was so sick for years, couple of years, because of taking this medicine that was helping me, plus I was taking all kinds of other medicines and gabapentin. […] I had a long slew of medications and I was just desperate to … because I was so rough. I was suicidal, it was all bad. I was desperate for anything. He suggested [cannabis].” (F3, V10)	*N* = 1, *n* = 1
**Outcomes from cannabis use**			
Theme 22: Some female veterans report inconsistent effects from cannabis use, which was not reported in male participants.			
22.1 Inconsistent effects reported by females	Female veterans report inconsistent effects from using cannabis.	“So, yeah, for me it’s the only thing is barely finding a way to understand how it can help me better, because it’s very sporadic. Sometimes it helps, sometimes it doesn’t.” (F4, V12)	*N* = 2, *n* = 2
Theme 23: One veteran reported cannabis interacting with other medications.			
23.1 Interactions with other medications	Veterans report having negative experiences with cannabis interacting with other medications.	“So I put in a vape and I started out like 6% THC and stuff like that, and I got up to 13% THC, and at the same time a medical condition, non-military-related one, I had new medication. Well there is a conflict between the CBD or the THC and this new medication. I ended up in hospital. So, like, this was me trying to figure out my doses.” (F2, V8)	*N* = 1, *n* = 1
**Facilitators of cannabis use**			
Theme 24: Some veterans report obtaining medicinal cannabis through online cannabis dispensaries.			
24.1 Online dispensaries	Veterans report ordering/receiving medical cannabis from private dispensaries.	“Like, I buy my stuff through Shoppers Drug Mart, which is absolutely a godsend because they’re really, really helpful. They’re kind and they will take a lot of time, especially some things when I need it because I’m just trying to navigate through this.” (F4, V11)	*N* = 1, *n* = 2
Theme 25: Some veterans report receiving cannabis-related information from cannabis companies and social media platforms.			
25.1 Cannabis companies	Veterans report that their primary source of information on cannabis comes from cannabis companies.	“I wish that I had met with the people from Spectrum initially, because they’re very knowledgeable. Like, the nurse practitioner I talked to, she’s so knowledgeable, she also knows my condition, which a lot of people are very uneducated about it. Most people are. So I really appreciate that. And just having that wealth of knowledge from them is really great, and I can phone them anytime and they will answer everything and anything.” (F3, V10)	*N* = 1, *n* = 1
25.2 Social media	Veterans report that their primary source of information on cannabis comes from social media.	“I’d like to have maybe like once a month or once a week somebody sends me a notification, ‘Did you try this?’ Like, I’m on some other cannabis pages on Facebook and they talk a lot about different things.” (F4, V11)	*N* = 1, *n* = 1
**Stigma around cannabis use**			
Theme 26: Some veterans report an absence of stigma around cannabis use among fellow veterans.			
26.1 Absence of stigma among fellow veterans	Veterans report not experiencing any stigma around cannabis use from their fellow veterans.	“Yeah, I don’t think there is any negative impact, either. Like, with the friends it’s the same. A lot of them—of my friends are retired people and they’re doing it for fun or some other people are doing it for pain. So it’s something that everybody is talking about, it’s like having a beer nowadays, really. Which is a good thing, in a way.” (F4, V11)	*N* = 1, *n* = 1
Theme 27: Some veterans report experiencing self-stigma (i.e., the internalization of systemic and public stigma around cannabis use) when purchasing cannabis from private medical cannabis clinics that they perceive as shady.			
27.1 Experience of self-stigma when buying cannabis from private medical cannabis clinics	Veterans report experiencing self-stigma when purchasing cannabis from private medical cannabis clinics they perceive as shady.	“I don’t like—with CannaConnect is to me it feels like it’s a shady drug deal behind, like, illegal drug deal or something because, you know, you have a doctor you’ve never met through video screen doesn’t know your history, I mean, and they’re just willing to write your prescription. It seems so, you know—it just kind of for me it plays on that stigma.” (F1, V2)	*N* = 1, *n* = 1
**Questions and concerns about cannabis use**			
Theme 28: Some veterans report having questions about how to transition from other medications to cannabis, the effects of various ratios of THC to CBD, as well as the timing of cannabis consumption.			
28.1 Transitioning from other medications to cannabis	Veterans have questions around how to transition from other medications to cannabis.	“With a treatment plan, weaning off of these, you know, terrible, terrible narcotics. I wanna see some more stuff like that. Safe transitioning centers like they’re doing for heroin in Vancouver. I mean, if there can—they can get that approved for a narcotic like heroin as a transitionary thing, I don’t see how we can have dispensaries and no treatment centers with THC CBD.” (F2, V7)	*N* = 1, *n* = 3
28.2 Amount of THC vs. CBD	Veterans have questions about the effects/consequences of different ratios of THC to CBD with respect to symptom management.	“Something I’m curious about is the THC/CBD relationship between the two and that you need to have a little bit of THC with the CBD—is that true or not? Or, like, do they need to be a bit of THC with the CBD to make it work?” (F4, V11)	*N* = 1, *n* = 3
28.3 Timing of consumption	Veterans have questions about when the best time to take cannabis is.	“I still would like to have one of you guys [health professional] tell me what to do. Take this in the morning, take this at bedtime. Like, I’d like to have just some clear direction.” (F4, V11)	*N* = 1, *n* = 1

^a^*N* = number of focus groups in which subtheme was endorsed; *n* = number of veterans who endorsed subthemes.

## Interpretation

This is one of few studies to qualitatively examine Canadian veterans’ views on and experiences with medicinal cannabis and the only study to date that has included views of male and female veterans.

### Reasons for Cannabis Use

Veterans experienced at least one medical condition for which medicinal cannabis was prescribed; however, it was clear that comorbidity was the norm, with most veterans experiencing concurrent medical conditions (e.g., chronic pain, musculoskeletal injury, insomnia, PTSD, depression, anxiety). For this reason, most veterans were taking multiple medications to manage their complex symptoms, which were often accompanied by negative side effects, particularly in the case of opioids and, to a lesser degree, gabapentin. In many cases, it was the quantity and severity of negative side effects from other medications that motivated the switch to medical cannabis. This aligns with qualitative work by Clary et al.^20^ on cannabis use among young U.S. military members and veterans who reported using cannabis as an alternative to opioid and benzodiazepine medications for the treatment of physical ailments. It also aligns with work by Croulet^19^ on perceptions of cannabis use among U.S. veterans who reported that cannabis has fewer side effects compared to pharmaceuticals and did not hinder any aspects of their life. In a study by Krediet et al.^36^ on experiences with medicinal cannabis in the treatment of PTSD in veterans from the Netherlands, all participants reported having fewer side effects using cannabis than with conventional medications. Baumbusch and Sloan Yip^37^ conducted a qualitative study among civilians to investigate new cannabis use among older adults (65 years or older) and observed that the main reasons for cannabis were pain management, as a sleep aide, and as an alternative to over-the-counter medications to avoid negative side effects associated with those medications.^37^

### Outcomes from Cannabis Use

Veterans reported beneficial outcomes from their switch to medical cannabis, including improvements in sleep quality, interpersonal relationships, mood, and quality of life. Several veterans interviewed replaced medications that targeted a single symptom with cannabis, which they believed had diffuse effects that improved multiple symptoms. This is not to say that medical cannabis is a side effect–free panacea. Some veterans reported experiencing dry mouth, throat or sinus irritation, increased appetite, and weight gain from cannabis use. These findings accord with qualitative studies on perceptions of the risks and benefits of medicinal cannabis use among U.S.^18–20,35^ and Dutch^35^ veterans that observed that most veterans perceived the benefits of cannabis use to outweigh the harms. This seems to conflict with a qualitative synthesis on cannabis use among military veterans by Turna and MacKillop,^14^ who cited evidence of associations between cannabis use and negative health outcomes, increased substance use, psychiatric disorders, and increased self-harm.

Self-reports of benefit from medicinal cannabis use in the absence of clear evidence raises the possibility of a placebo response. In fact, cannabis has been found to activate psychoneuroimmunological mechanisms shown to mediate placebo analgesia, which may explain why it is often used by individuals who experience chronic pain.^37,38^ Casarett^39^ argued that placebo-controlled trials of medicinal cannabis have been flawed by inadequate blinding, making them susceptible to the placebo response, potentially leading to an overestimate of the effectiveness of medical cannabis. Findings on the role of the placebo response with respect to cannabis use also tie into the perception of risk associated with its use. A qualitative study on the formation of risk perception around cannabis use in a sample of veterans receiving treatment for a substance use disorder identified that the most salient factor informing risk perception was an individual’s personal experience with cannabis.^18^ An important implication is that individuals who cannot identify any losses or negative experiences associated with their own cannabis use are less likely to consider potential harms. Considering this, it is worth exploring whether there is a selection bias in qualitative studies on cannabis use whereby individuals who have had positive experiences with cannabis are more likely to volunteer for such studies. Conversely, many studies may be conducted in treatment centers with a strong bias against the use of cannabis for medicinal purposes.^40,41^

### Facilitators of Cannabis Use

Several veterans commented on how efficient receiving coverage through the VAC for medical cannabis was, which is contrary to low support from medical doctors in Canada.^42,43^ This ease of receiving coverage was appreciated by many veterans and facilitated medical cannabis use, which is associated with the growth and cost of the VAC cannabis reimbursement program and the growing number of private medical cannabis clinics targeted toward veterans that have opened for business in recent years.^44^ This growth is perhaps at odds with the gaps in knowledge about medicinal use, including the lack of a clear indication of what dose and form of medicinal cannabis will be of benefit for veterans with different conditions^19^—questions that veterans would like answers to as well.

Many veterans reported positive experiences with private medical cannabis clinics because they help in navigating the reimbursement system, completing VAC paperwork, obtaining a prescription for medical cannabis, scheduling and coordinating health care services, and answering questions about cannabis.^45^ Some of these clinics also put on social activities for veterans, offering a sense of community as well as opportunities for peer support. A shortage of family physicians in many regions of the country is another reason why many veterans have gravitated toward these private clinics, because that may be the only way to obtain a prescription.^46^ These clinics are filling a gap in care and direction that was missing for veterans; however, consideration of the private, for-profit nature of these clinics is warranted. Some provincial medical colleges have expressed concern about physicians charging patients for consultation as well as the use of one-off telemedicine sessions in the absence of a thorough case review and assessment of need.^44,47^ Some of the veterans in our study reported being prescribed the maximum daily reimbursable dose of cannabis by physicians at these clinics in the absence of expressed need. This echoes a concern expressed by Kahan et al.^47^ that a lack of regulatory oversight of cannabis clinic protocols could result in patients being prescribed an excessive dose. Of course, the paucity of research on medicinal cannabis dosing for different conditions contributes to this problem, making it difficult for prescribers to know how much is indicated for a given patient with a given condition.^48,49^ Nevertheless, there have been efforts to establish safe practice guidelines for the use of medicinal cannabis.^50–54^

### Barriers to Cannabis Use

Several veterans reported past experiences of feeling intoxicated, anxious, or paranoid while using cannabis. The likelihood and severity of these side effects would be expected to vary with dose, ratio of THC to CBD, and method of consumption (e.g., smoked versus ingested). In many cases, veterans identified a dosage, strain, and method of consumption that minimized or avoided these side effects through their own process of trial and error. A lack of guidance around dosing of medicinal cannabis necessitating a trial-and-error approach by the user has been cited as a concern in numerous studies^35,48,49,55^ and was reported by Canadian veterans in our study. Reliance on unvetted sources of information and experimentation with dosing is a potentially dangerous approach that could result in overdosing and negative experiences with cannabis or underdosing and therefore failure to gain therapeutic benefit. Resolving confusion around dosage may help veterans avoid some of these potential pitfalls, though numerous challenges remain in establishing safe and accurate dosing information in the regulated cannabis market.^48,49^

Some of the barriers to medicinal cannabis use reported by veterans seem to relate to growing pains within the cannabis industry generally. For example, the difficulty in obtaining a stable supply has affected both the medicinal and recreational markets since legalization.^56,57^ These disruptions in supply have left many veterans having to switch providers, which can be troublesome because it is delayed by paperwork and approvals, resulting in a lapse of coverage. There may be scope for the VAC to streamline the process of switching providers to help ameliorate this challenge for veterans.

Restrictions to driving and travel were frequently reported barriers to medicinal cannabis use reported by veterans in this study. This aligns with a qualitative study of veterans in the Netherlands.^35^ In a survey of adult cannabis users in Canada (*N* = 1093), Wickens et al.^58^ observed that most believe that frequent recreational and medical cannabis users can drive safely after exposure to cannabis. This is despite at least three systematic reviews and meta-analyses that suggest that the acute use of cannabis increases collision risk.^59–61^ In a narrative review of the impact of cannabis on road safety, Brands et al.^62,64^ concluded that “‘cannabis is cannabis’ and it produces impairments in driving, regardless of whether it is used for recreational or medical purposes.”^(p5)^ This discrepancy between user perception and the literature is concerning given that a higher proportion of medical cannabis users engage in driving under the influence of cannabis, relative to recreational-only users.^58^ However, as pointed out by Turna and MacKillop,^14^ medical cannabis users may be more likely than recreational users to opt for products with lower THC levels, which could reduce the collision risk associated with use. Some of the veterans in this study reported that their daily medicinal cannabis use precluded them from being able to drive without fear of prosecution and therefore limited their ability to travel locally. Many veterans reported that despite a desire to travel outside of Canada, they have held back because they do not want to have to return to relying on other medications (e.g., opioids) for symptom management. Unfortunately, traveling with medicinal cannabis internationally will remain a challenge for the foreseeable future and is not a problem that can be rectified without an international shift in perceptions around cannabis use.

### Stigma around Cannabis Use

A frequently reported barrier experienced by veterans in this study was stigma around cannabis use. This echoes work by Gibson et al.^55^ who conducted a qualitative study on perceptions of alcohol and cannabis use among male Canadian veterans with PTSD (*N* = 5) and observed that participants hesitated to use cannabis, even when medically prescribed, because they were concerned about how they may be perceived by their peers and society at large. As observed by Compton et al.^63^ stereotypes and biases about cannabis users continue to persist despite a general increase in the acceptability of cannabis use within society. It is common for veterans to experience self-stigma around cannabis use,^35^ owing in part to deeply ingrained institutional military regulations and values prohibiting cannabis use.^55^ In a qualitative study on stigma around cannabis use among older veterans in the United States engaged in medicinal cannabis use (*N* = 32, 94% male, *M*_age_ = 71), Clary et al.^21^ identified three themes: (1) negative stereotypes regarding people who use cannabis, (2) negative portrayals of people who use cannabis in the media, and (3) hesitation in disclosing medicinal cannabis use to friends, family, and physicians. One quote from a participant in the Clary et al.^21^ study is illustrative: “Yeah, I know most people think oh yeah medical cannabis, just an excuse for people, to get you know, pot so they can get high.”^(p4)^ The fear of disclosing medicinal cannabis use could be isolating, particularly for veterans using it to manage symptoms associated with chronic pain or PTSD, with potential consequences to their health and well-being.^21^ Even in the Canadian context where medicinal cannabis has been legal for more than a decade, social norms around medicinal use remain unfavorable for many populations.^42^ Of note, there is less stigma around the use of cannabis oil as compared to smoking,^35^ and media portrayals of cannabis users are becoming more positive,^21^ signaling that stigma may become less of an issue over time.

### Questions and Concerns about Cannabis Use

Another frequently reported barrier experienced by veterans in this study was a lack of scientific medical knowledge around cannabis use among medical practitioners and within the medical system generally. In a qualitative study on medicinal cannabis use among community-dwelling older Canadian adults (*N* = 12, age 65+), Baumbusch and Sloan Yip^36^ observed that older adults received little guidance from their family physician and relied on information from other sources, such as friends, cannabis store staff, and the media. Several reasons have been proposed for why many physicians are reluctant to prescribe cannabis and, even when they prescribe, offer little guidance to their patients. According to Ng et al.,^57^ lingering stigma within health care, a lack of training and resources, and insufficient clinical evidence may all contribute to a physician’s hesitancy to prescribe cannabis. This is coupled with many physicians resenting that the government has made them the gatekeepers to what they consider to be an unproven drug.^44^ A related barrier is the growing shortage of family physicians in Canada—which means reduced access to care for all Canadians, veterans included.^46^

### Limitations and Recommendations for Further Research

This was a qualitative investigation and cannot speak to the causal relationship between factors. As in any study reliant on participant self-report, the validity of the information collected in this study is limited by the accuracy of each veteran’s self-report. The small sample size (*N* = 12) and limited regional, ethnic, and racial diversity of the sample may reduce the generalizability of the findings to the wider veteran population. Unfortunately, limited sociodemographic information was collected for each participant. This was determined to be an acceptable trade-off for this study given the nature of the focus groups and the desire to offer participants confidentiality. We did not engage in member-checking of our thematic analysis, which limits its trustworthiness. Given the qualitative nature of the study, we were unable to determine the prevalence or relative importance of the various issues brought up; however, this study provides valuable information for future research in this area.

Future research should further explore cannabis use behaviors, reasons for cannabis use, and outcomes from cannabis use, as well as the relative impact of each of the identified facilitators and barriers to cannabis use among a larger and more diverse sample of Canadian veterans. Rigorous peer-reviewed research is needed to identify what dose, strain, form, and schedule of administration is best suited for which symptoms under which conditions. It is also important to determine the potential long-term effects of medicinal cannabis use, something that concerns many veterans who rely on medical cannabis for daily symptom management. Lastly, more research is needed to understand the roles that social, structural, and self-stigma play in veterans’ experiences around medicinal cannabis use, and to what degree legalization of the recreational market has impacted experiences of stigma.

## Conclusion

The veterans included in this study experienced a range of health conditions and reported a history of using multiple medications to treat those conditions, often experiencing negative side effects. Being able to replace multiple medications with cannabis was reported to result in markedly beneficial outcomes for most participants, leading to fewer adverse effects. That said, a lack of guidance surrounding dosing remains a concern for veterans. Disruptions in supply, difficulty switching providers, restrictions on driving and travel, stigma, and a lack of scientific medical knowledge around cannabis presented ongoing challenges to veterans. The expansion of the VAC medical cannabis program coupled with the ease of receiving coverage has been accompanied by the growth of a private medical cannabis industry serving the needs of veterans. Most of the veterans in this study reported being happy with the VAC reimbursement program, as well as the service they have received from private clinics. Concern remains around the discrepancy between qualitative reports of beneficial outcomes from medicinal cannabis use and equivocal findings around the benefit-to-harm ratio in the wider literature. Currently, the VAC reimbursement program remains challenged by unclear indication for which veterans, with what condition(s), at what dose, and in what form medical cannabis is most beneficial. Future research is needed to help answer these questions to ensure that the reimbursement program remains viable and veterans receive safe and effective care.

## Figures and Tables

**Figure:**
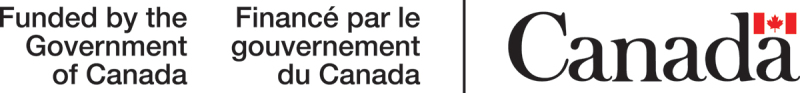


**Figure:**
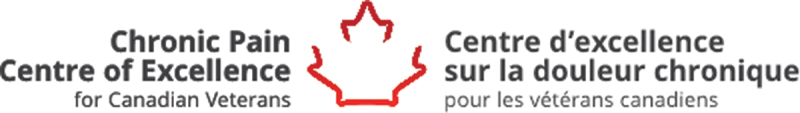


## Data Availability

The data that support the findings of this study are available on request from the corresponding author, JR. The data are not publicly available because they contain information that could compromise the privacy of research participants.

## Appendix

**Table:** Focus group guide.

Part 1: Welcome and introductions 15 min	Facilitators will introduce themselves and give a quick briefing on the project, its stakeholders, and the end goals. Participants will take a few minutes to introduce themselves and share a bit about themselves. “Tell us your name, a bit about your military background, and what you like to do for fun.”
Part 2: Contextual inquiry 60 min	As “prework”, veterans will be asked to reflect on a series of questions, making note of their answers and taking photographs and collecting artifacts where appropriate to help illustrate their experiences. When do you use cannabis? Why do you use cannabis? How does cannabis make you feel? How does cannabis impact your life? In this first activity, we will ask veterans, one-by-one to share their “prework.” After each individual’s presentation, we will ask the group if they have any comments, questions, or reflections. We will probe with: Does “x’s” experience resonate with you? Did you find anything surprising?
Part 3: Veteran-centered research 40 min	Facilitators will then lead participants on a second activity where we will narrow the focus to uncover the research questions that veterans are most interested in getting answers to. We will begin by giving each veteran some time to reflect on the following questions and jot down notes: Reflecting on your individual experience, what do you wish you better understood or knew more about, with respect to cannabis and cannabis use? Consider: How to use it. How others use it. Its impact on various facets of your life. Its side effects. Other things that are important to you that you want to understand. We will then ask each veteran to share their answers, capturing their ideas on Post-its and affinity mapping them on a virtual whiteboard. Once each veteran has shared, we will reflect on the themes that have arisen and probe deeper to fill in gaps and flesh out thematic areas where necessary.
Part 4: Reflect and close 5 min	Facilitators will give the group time to reflect on their experience in the session and make any final comments. Facilitators will then outline next steps in this project and help participants to understand how their participation will propel this research project forward.
